# VEGF Receptor 1-Expressing Macrophages Recruited from Bone Marrow Enhances Angiogenesis in Endometrial Tissues

**DOI:** 10.1038/s41598-019-43185-8

**Published:** 2019-05-07

**Authors:** Kazuki Sekiguchi, Yoshiya Ito, Kyoko Hattori, Tomoyoshi Inoue, Kanako Hosono, Masako Honda, Akiko Numao, Hideki Amano, Masabumi Shibuya, Nobuya Unno, Masataka Majima

**Affiliations:** 1 Department of Pharmacology, Kitasato University School of Medicine, Sagamihara, Kanagawa Japan; 2 Department of Molecular Pharmacology, Graduate School of Medical Sciences, Kitasato University, Sagamihara, Kanagawa Japan; 3 Department of Obstetrics and Gynecology, Kitasato University School of Medicine, Sagamihara, Kanagawa Japan; 4 Gakubunkan Institute of Physiology and Medicine, Jobu University, Takasaki, Gunma Japan

**Keywords:** Experimental models of disease, Cell signalling

## Abstract

Angiogenesis is critical in maintenance of endometrial tissues. Here, we examined the role of VEGF receptor 1 (VEGFR1) signaling in angiogenesis and tissue growth in an endometriosis model. Endometrial fragments were implanted into the peritoneal wall of mice, and endometrial tissue growth and microvessel density (MVD) were determined. Endometrial fragments from wild-type (WT) mice grew slowly with increased angiogenesis determined by CD31^+^ MVD, peaking on Day 14. When tissues from WT mice were transplanted into VEGFR1 tyrosine kinase-knockout mice, implant growth and angiogenesis were suppressed on Day 14 compared with growth of WT implants in a WT host. The blood vessels in the implants were not derived from the host peritoneum. Immunostaining for VEGFR1 suggested that high numbers of VEGFR1^+^ cells such as macrophages were infiltrated into the endometrial tissues. When macrophages were deleted with Clophosome N, both endometrial tissue growth and angiogenesis were significantly suppressed. Bone marrow chimera experiments revealed that growth and angiogenesis in endometrial implants were promoted by host bone marrow-derived VEGFR1^+^/CD11b^+^ macrophages that accumulated in the implants, and secreted basic fibroblast growth factor (bFGF). A FGF receptor kinase inhibitor, PD173047 significantly reduced size of endometrial tissues and angiogenesis. VEGFR1 signaling in host-derived cells is crucial for growth and angiogenesis in endometrial tissue. Thus, VEGFR1 blockade is a potential treatment for endometriosis.

## Introduction

Endometriosis, characterized by extra-uterine growth of endometrial tissue, is a common gynecological disease in women of reproductive age^1^. The most common symptoms are pelvic pain and infertility, both of which have an adverse effect on quality of life^2,3^. Endometriosis is highly dependent on angiogenesis^4^ (the formation of new blood vessels from pre-existing vessels), which is critical for both normal development and homeostasis as well as for certain pathological conditions^5,6^. There are numerous endogenous factors that regulate angiogenesis; however, vascular endothelial growth factor (VEGF) and its receptors are the prime regulators of both physiological and pathological angiogenesis^7,8^. The VEGF pathway plays a critical role in ischemic angiogenesis and tumor growth via receptor signaling-dependent mechanisms^9–11^. The most common isoform of VEGF, VEGF-A, binds to two receptor tyrosine kinases: VEGF receptor 1 (VEGFR1) and VEGF receptor 2 (VEGFR2). VEGFR2 is expressed mainly by endothelial cells, whereas VEGFR1 is also expressed by hematopoietic stem cells and inflammatory cells, such as monocytes and macrophages, and regulates chemotaxis^12–14^. VEGFR1 binds VEGF-A with an affinity approximately ten times that of VEGFR2; however, the underlying biological mechanism is not fully understood. VEGFR2-null mice fail to develop blood vessels and die *in utero*, indicating that VEGFR2 signaling is essential for development of the vascular system^15^. By contrast, VEGFR1-null mice exhibit overgrowth and disorganization of blood vessels, suggesting that VEGFR1 is a negative regulator of angiogenesis during embryonic development.

However, when we generated transgenic mice expressing a variant of VEGFR1 that lacks the tyrosine kinase domain (VEGFR1TK^−/−^), the mice appeared healthy and showed normal blood vessel formation^16^. Expression of VEGF and VEGF receptors (VEGFRs) increases during the healing of wounds and gastric ulcers and during recovery from ischemia^17,18^. Indeed, we recently showed that VEGFR1 signaling facilitates angiogenesis during recovery from ischemia and gastric ulcers^17,18^. Under these pathological conditions, VEGFR1-expressing cells increase angiogenesis, leading to speedier recovery from ischemia and tissue damage.

We previously reported that endogenous prostaglandin (PG) E_2_ plays a role in the growth of endometrial tissue and angiogenesis in a mouse transplantation model by inducing VEGF production^19^. We also found that signaling via PGE_2_ increased VEGF-dependent angiogenesis during chronic inflammation, and in the tumor microenvironment^10,20–22^. PG receptor signaling-mediated increases in cAMP levels facilitate both angiogenesis and VEGF production^23^. Further, we found that VEGF neutralizing antibody treatment reduced the growth of endometrial tissue and angiogenesis in a mouse transplantation model^24^. The implantation of endometrial tissues isolated from wild type (WT) mice to the peritoneal cavity in VEGFR1TK^−/−^ mice showed reduced angiogenesis and growth of implants, suggesting that host VEGFR1 signaling is critical for the maintenance of implants^24^. However, it remains unknown how VEGFR1 signaling regulates angiogenesis and development of endometriosis.

In the present study, we clarified that VEGFR1 signaling in host-derived cells, especially CD11b^+^ macrophages plays a role in growth and angiogenesis in endometrial tissues. This study suggests that blocking VEGFR1 with antibodies or small molecule kinase inhibitors may become a promising option to the treatment of endometriosis.

## Materials and Methods

### Animals

Eight-week-old female C57BL/6 wild-type (WT) mice were purchased from CLEA Japan (Tokyo) and used as controls in experiments involving 8-week-old female VEGFR1TK^−/−^, which were developed previously (Recombinant DNA Experiment Approve Number 3937)^16^. The knockout mice were backcrossed to a C57BL/6 background for more than ten generations. Green fluorescent protein transgenic C57BL/6 (GFP^+^TG) mice were also generated in-house (Recombinant DNA Experiment Approve Number 3937). TK^−/−^ mice and GFP^+^TG mice were crossed to obtain GFP^+/+^TK^−/−^ mice (GFP^+^TK^−/−^ TG)^17^. All mice were housed in a limited access animal facility with a temperature maintained at 25 ± 1 °C and relative humidity at 60 ± 5%. A 14 h light/10 h dark (6 AM to 8 PM) cycle was established using artificial lighting. All experimental procedures were approved by the Animal Experimentation and Ethics Committee of the Kitasato University School of Medicine (1114, 2015–022), and were performed in accordance with the guidelines for animal experiments set down by the Kitasato University School of Medicine, which are in accordance with the “Guidelines for Proper Conduct of Animal Experiments” published by the Science Council of Japan. Mice used for survival studies were examined by animal care takers and the overall health status was checked by trained professionals. Mice were euthanized by pentobarbital sodium when they were found in a moribund state as identified by inability to maintain upright position and/or labored breathing. The mice for *in vivo* experiments were constantly checked daily throughout the experiment periods. Drugs were given under inhalation anesthesia with isoflurane. Tissue collection procedures were performed under anesthesia with pentobarbital sodium. At the end of the experiments, the animals were euthanized by exsanguination under anesthesia with pentobarbital sodium followed by cervical dislocation.

### Bone marrow transplantation

Bone marrow transplantation was performed as previously described^25^. Briefly, donor bone marrow cells were harvested from GFP^+^TG or GFP^+^TK^−/−^ TG mice, bone marrow mononuclear cells were isolated by filtration through nylon mesh filter, and the mononuclear cells were transplanted into irradiated WT mice via the tail vein. GFP^+^TG bone marrow-transplanted mice were named GFP^+^WT BM chimeric (BMC) mice (n = 12). GFP^+^TK^−/−^ TG bone marrow-transplanted mice were named GFP^+^TK^−/−^ BMC mice (n = 12). After 6–8 weeks of bone marrow transplantation, peripheral blood from mice was collected via tail vein. Mononuclear cells were obtained from whole blood by Lymphosepar II (Immuno-Biological Laboratories, Fujioka). FACS analysis for the peripheral leukocytes was performed on FACS Calibur (BD Biosciences, Franklin Lakes, NJ, USA). Mice in which more than approximately 90% of the peripheral leukocytes were GFP-positive were used for the experiments.

### Endometrial transplantation model

**Figure 1:**
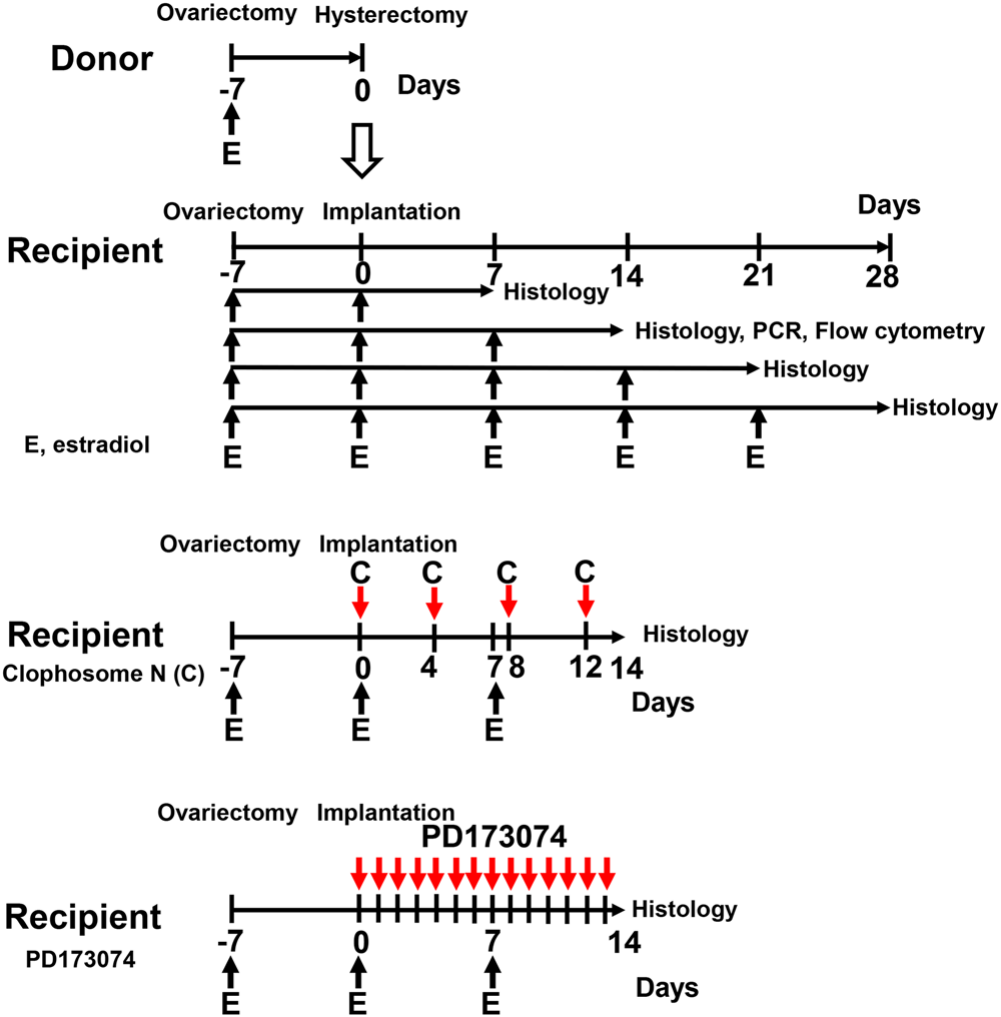
Experimental protocols for experimental endometriosis. Both donor and recipient mice were treated with estradiol (E). In some experiment, recipient mice were treated with Clophosome N (C) or PD173074. Tissue samples for analyses were collected at the indicated time.

Endometrial transplantation was performed as previously described (Fig. 1)^24,26^. Briefly, donor and recipient mice were bilaterally ovariectomized through paravertebral incisions to exclude endogenous estrogen and menstrual cycle. All donor and recipient mice received subcutaneous (s.c.) injections of estradiol dipropionate (100 mg/kg) in sesame oil (Obahormone depot; Aska, Tokyo) every week from the time of ovariectomy^24,27^. Seven days after ovariectomy, the uterine horns from the donor were removed, trimmed of connective tissue, and opened longitudinally in a tissue culture dish containing Dulbecco’s modified Eagle’s medium F-10 (Gibco, Grand Island, NY) at 37 °C, supplemented with 100 U/mL penicillin and 100 mg/mL streptomycin (Gibco, Grand Island, NY). Four round endometrial fragments (3 mm in diameter), which include the myometrium, were collected using a biopsy punch (Kai medical, Japan). The endometrial tissues were transplanted to the peritoneal wall of recipient mice with a 7-0 polypropylene suture (Ethicon, Johnson & Johnson, Japan), as described previously (Fig. 1)^24,26^; this location was chosen because it is in contact with the endometrial surface epithelium of the implants and peritoneum. Endometrial fragments from WT or TK^−/−^ mice were implanted ectopically into the peritoneum of either WT or TK^−/−^ mice. The wound was closed with a 3-0 suture and mice were placed on a warming carpet to prevent hypothermia. The day of implantation was defined as Day 0, and mice were euthanized under anesthesia on Days 7, 14, 21, or 28 post-implantation. The endometrial implants were removed and captured by taking digital photographs.

The captured digital images were uploaded to a computer and opened with ImageJ image analysis software. The implant outline was defined from the photographic image. Following tracing, the areas of the implants were calculated by ImageJ image analysis software. The results were expressed as the size of the implants per mm^2^. The four implants obtained from an individual recipient mouse were randomly assigned to experimental analyses; One of them was prepared for gene expression which examined by real-time reverse transcription-polymerase chain reaction (RT-PCR). The other one was used for immunohistochemistry. The rest of the two were prepared for immunofluorescence. All histological samples were first fixed in 4% formaldehyde in 0.1 M sodium phosphate buffer (pH 7.4) at 4 °C for 24 h for analyses. When implants from WT mice were transplanted into host WT mice, we expressed the transplants of WT; Implant → WT; Host combination as WT → WT. Using WT mice and TK^−/−^ mice, we created four different cross transplantation experimental groups; WT → WT (n = 13), TK^−/−^ → WT (n = 12), WT → TK^−/−^ (n = 12), and TK^−/−^ → TK^−/−^ (n = 12).

### Deletion of macrophages with Clophosome

Recipient mice were injected intraperitoneally (i.p.) with 0.7 mg of Clophosome N (F70101C-N; FormuMax Scientific, Palo Alto, CA, USA) per mouse (n = 4) or control liposomes (F70101-N) (n = 4) every four days starting at the Day 0 implantation (Fig. 1).

### Administration of an inhibitor of FGF

Recipient mice received an intraperitoneal (i.p.) injection of PD173047 (25 mg/kg/day, Selleck Chemicals, Houston, TX) every day for 2 weeks starting at the Day 0 implantation (n = 8 per group) (Fig. 1). Control mice received PBS (n = 8 per group). PD173047 is a selective inhibitor for FGF receptor 1 (FGFR1), and PD173047 also inhibits bFGF (FGF-2) induced cell growth and proliferation^28,29^

### Immunohistochemical analysis

After fixation in 4% formaldehyde, tissues were embedded in paraffin. Sections (3 μm thick) were cut using a sliding microtome and dewaxed in xylene, and endogenous peroxidases were quenched by incubation in 3% H_2_O_2_ buffer. Antigen retrieval was performed by heating sections in 0.01 M sodium citrate buffer (pH 6.0) in a microwave oven. The sections were then incubated at 4 °C overnight with a polyclonal rabbit anti-CD31 antibody (1:800; Ab28364; Abcam, Cambridge, MA, USA). After washing in phosphate buffer solution (PBS), sections were stained with conjugated secondary antibody (Histofine Simple Stain MAX PO; Nichirei Bioscience, Tokyo), washed again, and stained with DAB (dimethylaminoazobenzene) for approximately 2 minutes. Finally, sections were counterstained with Mayer’s hematoxylin. Control sections were treated with isotype-matched control IgG.

### Immunofluorescence analysis

Fixed samples of endometriotic lesions were then embedded in OCT compound (Sakura Finetek U.S.A., Inc., Torrance CA) and frozen at −80 °C before 8 μm sections were cut using a cryostat. The OCT compound was removed by washing in PBS, and the sections were incubated in 1% bovine serum albumin (BSA)/PBS at room temperature for 1 h overnight at 4 °C to block non-specific binding. Next, sections were incubated with the following primary antibodies at 4 °C overnight: polyclonal rabbit anti-VEGFR1 (1:200; abcam2350; Abcam, Cambridge, MA, USA), polyclonal goat anti-VEGFR1 (1:200; sc-316-g; Santa Cruz Biotechnology, Santa Cruz, CA, USA), polyclonal rabbit VEGF-A (1:100, ab46154; Abcam), monoclonal rat anti-CD31 (1:200; BD550274; BD Biosciences, Franklin Lakes, NJ, USA), monoclonal rat anti-CD11b (1:200; BD550282; BD Biosciences), polyclonal goat anti-S100A4 (1:200; TA318024; OriGene Technologies, Rockville, MD, USA), or polyclonal rabbit anti-bFGF (1:200; ab72316; Abcam). After washing in PBS, the sections were incubated with the following secondary antibodies (all at 1:200) for 1 h at room temperature: Alexa Fluor 488-conjugated donkey anti-rabbit IgG, Alexa Fluor 594-conjugated donkey anti-rabbit IgG, Alexa Fluor 594-conjugated donkey anti-rat IgG, Alexa Fluor 594-conjugated donkey anti-goat IgG, and/or Alexa Fluor 647-conjugated donkey anti-rabbit IgG. Control sections were incubated in isotype-matched controls for monoclonal antibodies. Images were observed and captured under a confocal scanning laser microscope (LSM710; Carl Zeiss, Jena, Germany; ×400 magnification) or a fluorescence microscope (Biozero BZ-9000; Keyence, Osaka; ×400 magnification)^30^. Positive cells were quantified randomly from 4 fields at ×400 magnification per mouse.

### Determination of vessel density

Microvessel density (MVD) in areas showing the most intense neovascularization (hot spots) within the endometrial implants was used as a measure of angiogenesis, as previously described^24,31^. Briefly, blood vessels in the ectopic endometrium were stained with an anti-CD31 antibody and areas showing the highest levels of neovascularization were identified by scanning the endometrial tissues at low power (×40 and ×100 magnification). Individual microvessels within the area of maximum neovascularization were counted in one ×400 field. We determined MVD in the peritoneum to muscle layer, which lies just below the endometrial implant, and in the distant peritoneum 5 mm from the peritoneum at which implants were transplanted. CD31^+^ endothelial cells were clearly differentiated from the adjacent microvessels, stromal cells, and other connective tissue elements. MVD was expressed as the mean of blood vessels in three high-power-fields (150 μm × 150 μm).

### Isolation of cells from implants

In another set of experiment, mice in the WT → WT (n = 4) and TK^−/−^ → TK^−/−^(n = 4) were anesthetized with pentobarbital sodium solution (60 mg/kg, i.p.), and the excised implants were placed immediately at room temperature in RPMI, minced into small pieces using scissors, and incubated in RPMI containing 0.05% collagenase (Type IV; Sigma Chemical Co., St. Louis, MO, USA) at 37 °C for 20 min. The tissue was then pressed through a 70 μm cell strainer. The cells were centrifuged at 2600 rpm for 10 min at 4 °C, and pelleted cells were resuspended in PBS. Leukocytes were isolated from the homogenates by density-gradient centrifugation on 33% Percoll™ (GE Healthcare Life Sciences, Piscataway, NJ, USA), as previously reported^32^. Non-parenchymal cells were collected from the interface between the 33% and 66% Percoll™ density cushions and centrifuged at 2700 rpm for 30 min at 4 °C. Viable, nucleated cells were counted by trypan blue exclusion and diluted to a uniform cell density.

### Flow cytometry analysis

Cells were incubated with the 2.4G2 mAb (anti-cγRIII/II) to block non-specific binding of the primary mAb. Then, cells were stained with a combination of the following fluorochrome-conjugated antibodies: anti-CD11b (clone M1/70, BioLegend, San Diego, CA, USA), anti-CD34 (clone MEC14.7, BioLegend) and anti-CD133 (clone 315-2C11, BioLegend). Samples were measured on a FACSVerse™ (BD, Franklin Lakes, NJ, USA). The data were analyzed using Kaluza software v1.3 (Beckman Coulter, Brea, CA, USA)^33^.

### Quantitative real-time RT-PCR analysis

Total RNA was isolated from endometriotic tissues using TRIzol reagent (Life Technologies, Grand Island, NY, USA), according to the manufacturer’s instructions. RT-PCR and real-time PCR were performed to measure CD31, VEGF-A, bFGF, cTGF, EGF, TGF-ß, Ang-1, Ang-2, and human glyceraldehyde-3-phosphate dehydrogenase (GAPDH) mRNA expression, as previously described^34^.

The following primer sequences were used:

CD31, 5′-CAGAGCCAGCAGTATGAGGAC-3′ (forward) and 5′-GCAACTATTAAGGTGGCGATG-3′ (reverse);

VEGF-A, 5′-ACGACAGAAGGAGAGCAGAAG-3′ (forward) and 5′-ATGTCCACCAGGGTCTCAATC-3′ (reverse);

bFGF, 5′-GGCTGCTGGCTTCTAAGTGTG-3′ (forward) and 5′-TTCCGTGACCGGTAAGTATTG-3′ (reverse);

CTGF, 5′-AACCGGGGAGGGAAATTATAG-3′ (forward) and 5′-TGGAATCAGAATGGTCAGAGG-3′ (reverse);

EGF, 5′-ATGGGAAACAATGTCACGAAC-3′ (forward) and 5′-CATCTCTCCCAAGCACTGAAC-3′ (reverse);

TGF-ß, 5′-TGTATTCCGTCTCCTTGGTTC-3′ (forward) and 5′-AACAATTCCTGGCGTTACCTT-3′ (reverse);

Ang-1, 5′-TGAAGGAGGAGAAAGAAAACC-3′ (forward) and 5′-GGATGCTGTTGTTGTTGGTAG-3′ (reverse);

Ang-2, 5′-TACACACTGACCTTCCCCAAC-3′ (forward) and 5′-AGTCCACACTGCCATCTTCTC-3′ (reverse); and

GAPDH, 5′-ACATCAAGAAGGTGGTGAAGC-3′ (forward) and 5′-AAGGTGGAAGAGTGGGAGTTG-3′ (reverse).

### Cell culture

Bone marrow-cells were isolated from the femur and tibia of 8-week-old WT mice (n = 4) and TK^−/−^ mice (n = 4)^32^. Femurs and tibias of mice were flushed with PBS, and erythrocytes were lysed by treatment with RBC lysis buffer (BioLegend). For the generation of bone marrow-derived macrophages, bone marrow cells were cultured in RPMI 1640 medium containing 10% fetal calf serum and macrophage colony stimulating factor (M-CSF) (20 ng/ml, BioLegend) plated in 6-well plates (1.0 × 10^6^ cells per well). At day 7, cells were either left untreated or treated with recombinant murine placental growth factor (PlGF) (BioVision, Inc., CA, USA) in RPMI 1640 medium for 6 hours. Bone marrow-derived macrophages were then harvested and homogenized in TRIzol (Life Technologies), and mRNA levels were measured by real-time RT-PCR.

### Data analyses

Data were expressed as the mean ± standard error of the mean (SEM). All statistical analyses were performed using JMP 10 (SAS Institute, Cary, NC, USA). For statistical evaluations a normality test and a variance test were done. Data that were normally distributed were analyzed using the parametric tests. Comparisons between the two groups were performed using Student’s *t*-test. One-way analysis of variance (ANOVA) followed by Tukey-Kramer post-hoc test was used to compare data among multiple groups. Student’s t-tests were applied to the analyses for the origin of the vasculature, pro-angiogenic factors, treatment with chlophosome, PD173047, cultured cells response, and flow cytometry. The changes in size and angiogenesis in the endometrial implants were compared with one-way ANOVA with Tukey-Kramer post-hoc test. A P value < 0.05 was considered statistically significant. The data for PCR and histology were collected from one implant per mice. The sizes of multiple implants obtained from an individual mouse were averaged, and the averaged value per mice was compared for analysis. For analysis of flow cytometry, we collected four endometrial implants from an individual mouse, and combined into a single sample.

## Results

### Host VEGFR1 signaling is critical in maintenance of endometrial tissues and angiogenesis

When WT endometrial fragments were implanted into estrogen-stimulated WT mice, the implanted endometrial tissues grew gradually. Growth peaked at Day 14 post-implantation (Day 0: 6.56 ± 0.18 mm^2^ *vs*. Day 14: 10.07 ± 0.51 mm^2^, P = 0.0029), and the size of the implants decreased thereafter (Day 21: 9.21 ± 0.54 mm^2^, Day 28: 7.92 ± 0.72 mm^2^) (Supplementary Fig. S1a,b). When we stained the endometrial tissues with an anti-CD31 antibody, we found that the density of neovascularized blood vessels in transplanted tissues at Day 14 was higher than that in naïve endometrial tissues (Day 0: 5.39 ± 0.31/2.25 × 10^4^ μm^2^, Day 14: 6.95 ± 0.34/2.25 × 10^4^ μm^2^, P = 0.025) (Supplementary Fig. S1c,d). These results were essentially the same as those in our previous report^24^.

**Figure 2:**
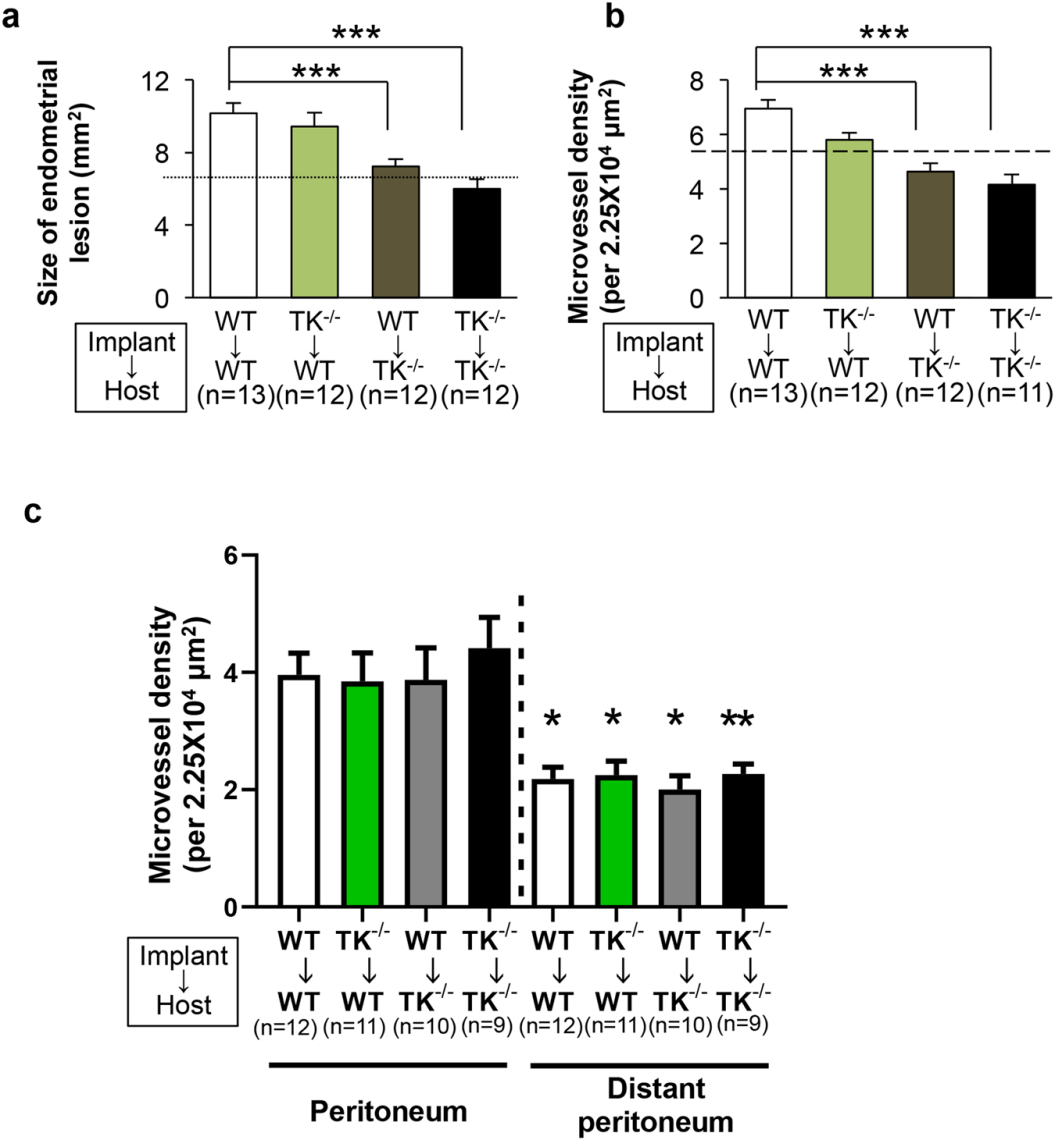
Host VEGFR1 signaling plays a role in growth in endometrial tissues and angiogenesis (**a**) Size of endometrial implants and (**b**) microvessel density at Day 14. *Dotted line* denotes the mean size of the endometrial lesion and the mean microvessel density from the WT → WT at Day 0. Data are expressed as the mean ± SEM (n = 11‒13 mice). ***P < 0.001 (one-way ANOVA) in comparison with WT → WT at Day 14.(**c**) Microvessel density in the perimetrium to muscle layer, which lies just below the endometrial implant, and in the distant peritoneum at Day 14. Data are expressed as the mean ± SEM (n = 9‒12 mice). *P < 0.05, **P < 0.01 (one-way ANOVA).

To estimate the role of host VEGFR1 signaling, we implanted WT or TK^−/−^ endometrial tissues into the peritoneal cavities of WT or TK^−/−^ mice (Fig. 2a,b). When TK^−/−^ endometrial fragments were implanted into the WT peritoneal cavity (TK^−/−^ → WT), the growth of the implants at Day 14 was not different from that of WT → WT (TK^−/−^ → WT, 9.43 ± 0.75 mm^2^ *vs*. WT → WT, 10.16 ± 0.55 mm^2^, P = 0.80) (Fig. 2a). By contrast, the WT → TK^−/−^ led to significant growth suppression at Day 14 when compared with the WT → WT (WT → TK^−/−^, 7.23 ± 0.42 mm^2^ *vs*. WT → WT, 10.16 ± 0.55 mm^2^, P = 0.004) (Fig. 2a). Similar results were observed with the TK^−/−^ → TK^−/−^ (TK^−/−^ → TK^−/−^, 5.99 ± 0.55 mm^2^ *vs*. WT → WT, 10.16 ± 0.55 mm^2^, P < 0.0001) (Fig. 2a). We confirmed our previous findings^24^, and suggested that the growth of endometrial fragments in estrogen-stimulated mice was promoted by host VEGFR1 signaling.

When the number of CD31^+^ vessels in the endometrial tissue implants were counted^24^, the density of CD31^+^ vessels in the WT → WT increased over time (Supplementary Fig. S1d), suggesting that angiogenesis was induced. At Day 14, we found that angiogenesis in the implants in the TK^−/−^ → WT was similar to that in the implants in the WT → WT (WT → TK^−/−^, 5.80 ± 0.27/2.25 × 10^4^ μm^2^ *vs*. WT → WT, 6.95 ± 0.34/2.25 × 10^4^ μm^2^, P = 0.06) (Fig. 2b); however, angiogenesis in the WT → TK^−/−^ was significantly lower than that in the implants in the WT → WT (WT → TK^−/−^, 4.63 ± 0.30/2.25 × 10^4^ μm^2^ *vs*. WT → WT, 6.95 ± 0.34/2.25 × 10^4^ μm^2^, P < 0.0001) (Fig. 2b). The same results were observed for the TK^−/−^ → TK^−/−^ (Fig. 2b). These results were essentially the same as the previous report^24^. This suggests that host-derived VEGFR1-expressing cells/tissues induce proangiogenic responses in implanted endometrial tissues.

### Origin of the vasculature in endometrial implants

As mentioned above, signaling via host-derived VEGFR1 is critical for the growth of endometrial tissues and for neovascularization of endometrial implants. Therefore, we next examined angiogenic responses in the parietal peritoneum that made contact with the implants, since angiogenesis in the parietal peritoneum may increase the growth of endometrial tissues by increasing the supply of oxygen and nutrients. Measurement of MVD in the parietal peritoneum at Day 14 post-implantation (Fig. 2c) revealed that vessel density in the WT → TK^−/−^ (3.87 ± 0.55/2.25 × 10^4^ μm^2^), TK^−/−^ → WT (3.84 ± 0.48/2.25 × 10^4^ μm^2^), and TK^−/−^ → TK^−/−^ (4.41 ± 0.53/2.25 × 10^4^ μm^2^) was similar to that in the WT → WT (3.96 ± 0.37/2.25 × 10^4^ μm^2^). In addition, MVD in the distant peritoneum from the parietal peritoneum of mice bearing endometrial implants (WT → WT, 2.18 ± 0.19/2.25 × 10^4^ μm^2^; TK^−/−^ → WT, 2.25 ± 0.24/2.25 × 10^4^ μm^2^; WT → TK^−/−^, 2.00 ± 0.24/2.25 × 10^4^ μm^2^; TK^−/−^ → TK^−/−^, 2.23 ± 0.17/2.25 × 10^4^ μm^2^) was lower than that in the parietal peritoneum just below the implants (*vs*. WT → WT, 3.96 ± 0.37/2.25 × 10^4^ μm^2^, P = 0.012; TK^−/−^ → WT, 3.84 ± 0.48/2.25 × 10^4^ μm^2^, P = 0.049; WT → TK^−/−^, 3.87 ± 0.55/2.25 × 10^4^ μm^2^, P = 0.024; TK^−/−^ → TK^−/−^, 4.41 ± 0.53/2.25 × 10^4^ μm^2^, P = 0.071) (Fig. 2c). These suggest that angiogenic responses in the parietal peritoneum were independent of VEGFR1.

**Figure 3:**
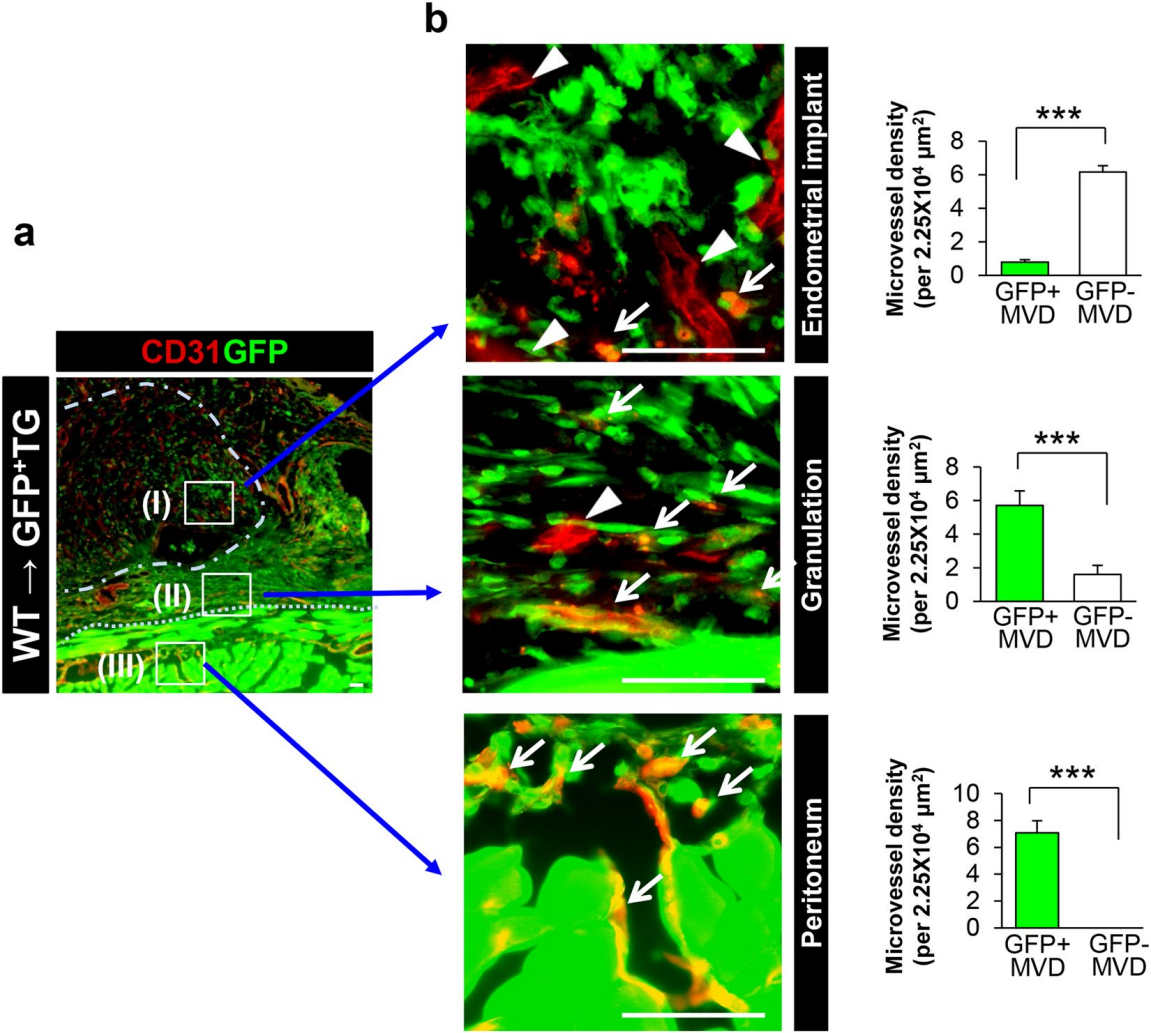
Origin of the vasculature in endometrial tissues (**a**) Immunostaining of CD31 (red) in WT → GFP^+^TG (green) mice. Endometrial implants (I), granulation tissues formed between the implant and the peritoneum (II), and peritoneum in contact with the endometrial implants (III) in WT → GFP^+^TG (green) at Day 14 post-implantation. The dotted line indicates the border between the peritoneum and granulation tissue. The dashed line indicates the border between the implant tissue and the granulation tissue. Scale bar, 25 μm. (**b**) Microvessel density of GFP^+^/CD31^+^ and GFP^−^/CD31^+^ microvessels in lesions from WT → GFP^+^TG mice at Day 14 post-implantation. Microvessel density (MVD) was determined in each part of the field at higher magnification. Arrows (↓) indicate GFP^+^/CD31^+^ endothelial cells. Arrow heads (∇) indicate GFP^−^/CD31^+^ endothelial cells. Scale bars, 50 μm. Data are expressed as the mean ± SEM (n = 8 mice). ***P < 0.001 (Student’s *t*-test).

Therefore, we next examined the origin of the vessels in WT → GFP^+^ TG (Fig. 3a). CD31^+^ vessel-like structures were identified in the WT implant (box I, upper panels in low-power field), the granulation tissue that formed at the margins of the implants (box II, upper panels), and the host parietal peritoneum (box III, upper panels) at lower magnification. GFP imaging revealed that the host parietal peritoneum was strongly GFP^+^, and that some GFP^+^ cells had infiltrated areas I and III. This suggests that host-derived cells were infiltrated to the implants and granulation tissue formed at the margins of the implants. When we examined MVD in areas I, II, and III at higher magnification, we found that the CD31^+^ vessel-like structures in areas II and III were strongly GFP^+^ (areas II: GFP^+^ MVD, 5.72 ± 0.86/2.25 × 10^4^ μm^2^ *vs*. GFP^−^ MVD, 1.60 ± 0.54/2.25 × 10^4^ μm^2^, p = 0.0006, areas III: GFP^+^ MVD, 7.08 ± 0.90/2.25 × 10^4^ μm^2^ *vs*. GFP^−^ MVD, 0/2.25 × 10^4^ μm^2^, p < 0.0001) (Fig. 3b),whereas those in area I showed slightly GFP^+^ (areas I: GFP^+^ MVD, 0.79 ± 0.15/2.25 × 10^4^ μm^2^ *vs*. GFP^−^ MVD, 6.58 ± 0.40/2.25 × 10^4^ μm^2^, p < 0.0001) (Fig. 3b). These results suggest that the blood vessels in the implants were not derived from the host, and that few vessels were sprouting from host tissues, even though angiogenesis in the implants was modulated by host-derived VEGFR1.

When we examined expression of VEGFR1 in implants in the WT → WT (Supplementary Fig. S2), almost all (>97.5%) of VEGFR1-positive cells were positive for CD31, CD11b, and S1004A, a marker for fibroblasts^35^ (% of VEGFR1^+^ cells in CD31^+^ cells: 97.6 ± 0.9; % of VEGFR1^+^ cells in CD11b^+^ cells, 98.4 ± 0.7; % of VEGFR1^+^ cells in S100A4^+^ cells: 99.2 ± 0.3) (Supplementary Fig. S2). As mentioned above (Fig. 3a), since the CD31^+^ vessels in the implants may not be derived from the host, VEGFR1-expressing CD11b^+^ cells and/or S1004A^+^ cells may facilitate angiogenesis via VEGF.

### Bone marrow-derived VEGFR1^+^ cells facilitate both growth and angiogenesis in endometrial implants

As mentioned above, most CD11b/S100A4^+^ cells were also VEGFR1^+^. Therefore, we hypothesized that the VEGFR1^+^ cells that infiltrated into the implants were recruited from the bone marrow. To test this, we generated bone marrow chimera (BMC) mice and examined growth and angiogenesis in endometrial tissues.

**Figure 4:**
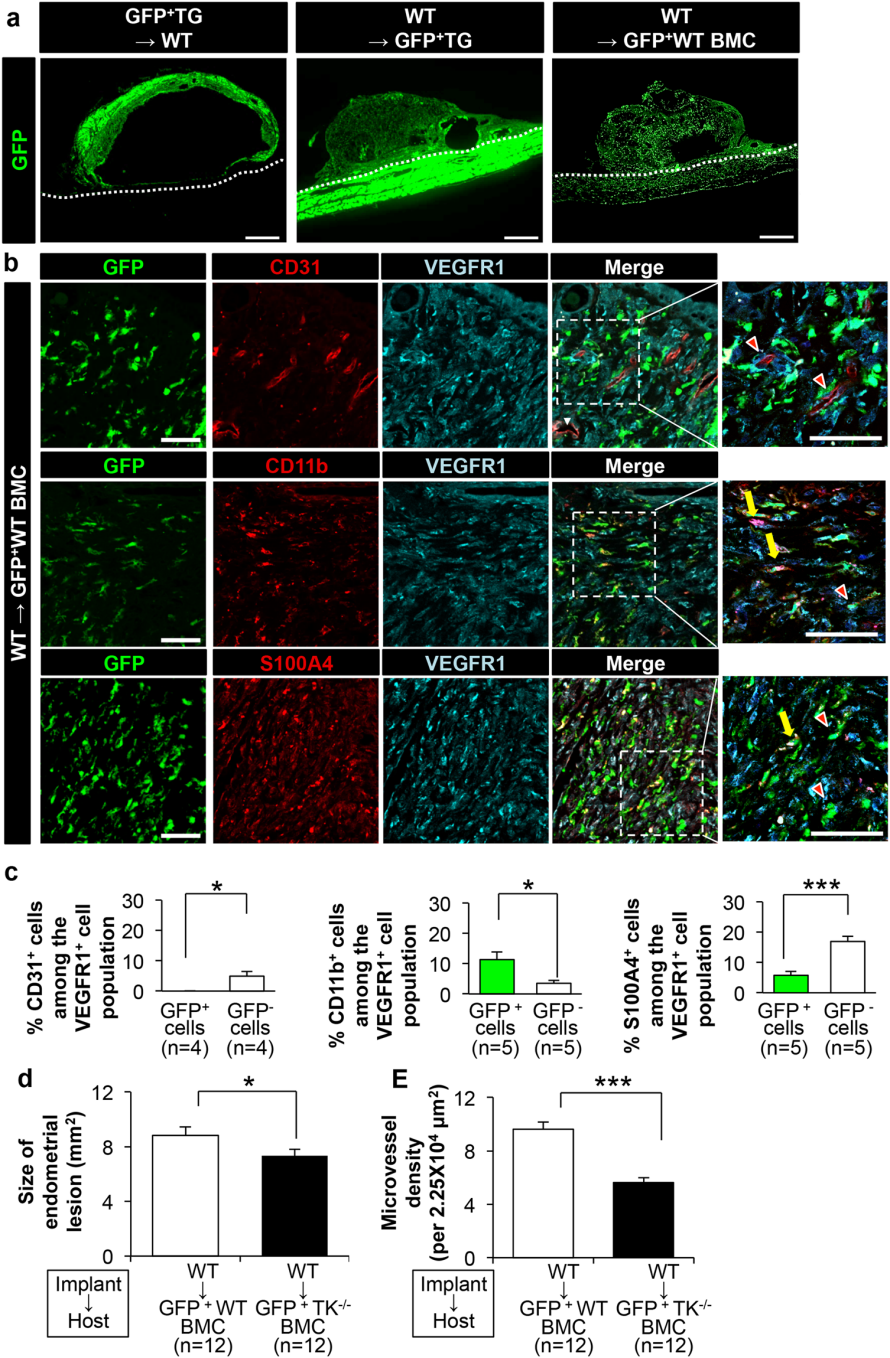
Bone marrow-derived cells accumulate in endometrial tissues at Day 14 post-implantation (**a**) GFP^+^ and GFP^−^ endometrial tissues implanted into mice. GFP^+^TG, GFP transgenic WT mice; GFP^+^WT BMC, GFP^+^ bone marrow chimera WT mice. Scale bars, 500 μm. (**b**) Recruitment of bone marrow-derived cells in GFP^+^ bone marrow chimera mice (GFP^+^WT BMC) receiving GFP^−^ WT implants. Arrow heads, GFP−VEGFR1+ cells; Arrows, GFP + VEGFR1+ cells. GFP^+^ cells, and CD31^+^, CD11b^+^, or S100A4^+^ cells, and VEGFR1^+^ cells in the endometrial implants. Scale bars, 50 μm. (**c**) Percentage of GFP^+^/CD31^+^, CD11b^+^, or S100A4^+^ cells, and GFP^−^/CD31^+^, CD11b^+^, or S100A4^+^ cells in the VEGFR1^+^ cell population. Data are expressed as the mean ± SEM (n = 4‒5 mice). *P < 0.05, **P < 0.01, and ***P < 0.001 (Student’s *t*-test t or † Welch’s test). (**d**,**e**) Size of endometrial implants (**d**) and microvessel density (**e**) in the WT → GFP^+^WT BMC and WT → GFP^+^TK^−/−^ BMC. GFP^+^WT BMC, GFP transgenic WT bone marrow chimera mice; GFP^+^TK^−/−^ BMC, GFP transgenic TK^−/−^ bone marrow chimera mice. Data are expressed as the mean ± SEM (n = 12 mice). *P < 0.05 and ***P < 0.001 (Student’s *t*-test).

When GFP^+^ WT endometrial tissues were implanted into WT mice, GFP^+^ cells were restricted to the implanted tissues and did not infiltrate the host parietal peritoneum (Fig. 4a, left panel). By contrast, when WT implants were implanted into GFP transgenic WT mice, a large number of GFP^+^ cells accumulated in the implants (Fig. 4a, middle panel). Transplantation of WT endometrial tissues into GFP transgenic WT BMC mice revealed that GFP^+^ cells accumulated in the implants (Fig. 4a, right panel). These results suggest that host cells, including bone marrow-derived cells, accumulate in the implants during growth and angiogenesis.

To examine the phenotype of the cells that accumulated in the implants, we next implanted non-GFP WT endometrial tissues into GFP transgenic WT BMC mice (WT → GFP^+^WT BMC). Tissues were then removed at Day 14 and stained with anti-CD31, anti-CD11b, and anti-S1004A antibodies (Fig. 4b). CD31^+^ vessel-like structures in the implants were also VEGFR1^+^; however, they were GFP^−^, suggesting that the blood vessels in the implants did not comprise bone marrow-derived cells (Fig. 4b). In addition, none of the cells were GFP^+^/CD31^+^/VEGFR1^+^ (% of GFP^+^/CD31^+^ cells among VEGFR1^+^ cells, 0 *vs*. % of GFP^−^/CD31^+^ cells among VEGFR1^+^ cells, 3.5 ± 1.1, P = 0.0265) (Fig. 4c). By contrast, most of the CD11b^+^ cells among the VEGFR1^+^ cell populations in the implants were GFP^+^ (% of GFP^+^/CD11b^+^ cells among VEGFR1^+^ cells, 11.3 ± 4.1 *vs*. % of GFP^−^/CD11b^+^ cells among VEGFR1^+^ cells, 3.5 ± 0.8, P = 0.0105) (Fig. 4c). When we stained implants with an anti-S1004A antibody, we observed accumulation of S1004A^+^/VEGFR1^+^ cells (Fig. 3b); however, the majority of these cells were GFP^−^ (% of GFP^+^/S100A4^+^ cells among VEGFR1^+^ cells, 16.9 ± 1.7 *vs*. % of GFP^−^/S100A4^+^ cells among VEGFR1^+^ cells, 5.8 ± 1.2, P = 0.0003) (Fig. 4c). Thus, a major population of GFP^+^ cells was CD11b^+^ rather than S1004A^+^ (Fig. 4b,c).

Further we examined the functional relevance of VEGFR1-expressing cells recruited from the bone marrow in terms of growth and angiogenesis in endometrial tissues (Fig. 4d,e). When we implanted WT endometrial tissues into TK^−/−^ BMC mice, the growth of the endometrial implants at Day 14 was more suppressed than that of WT implants in WT BMC mice (WT → GFP^+^WT BMC: 8.82 ± 0.60/2.25 × 10^4^ μm^2^ *vs*. WT → GFP^+^TK^−/−^ BMC: 7.34 ± 0.45/2.25 × 10^4^ μm^2^, P = 0.031) (Fig. 4d). The same was true for angiogenic responses (WT → GFP^+^WT BMC: 9.61 ± 0.53/2.25 × 10^4^ μm^2^ vs. WT → GFP^+^TK^−/−^ BMC: 5.61 ± 0.35/2.25 × 10^4^ μm^2^, P < 0.0001) (Fig. 4e). These results suggest that bone marrow-derived VEGFR1-expressing cells that accumulate in the implants facilitate both tissue growth and proangiogenic responses in endometrial fragments.

### Effect of macrophage deletion on angiogenesis in endometrial tissues

**Figure 5:**
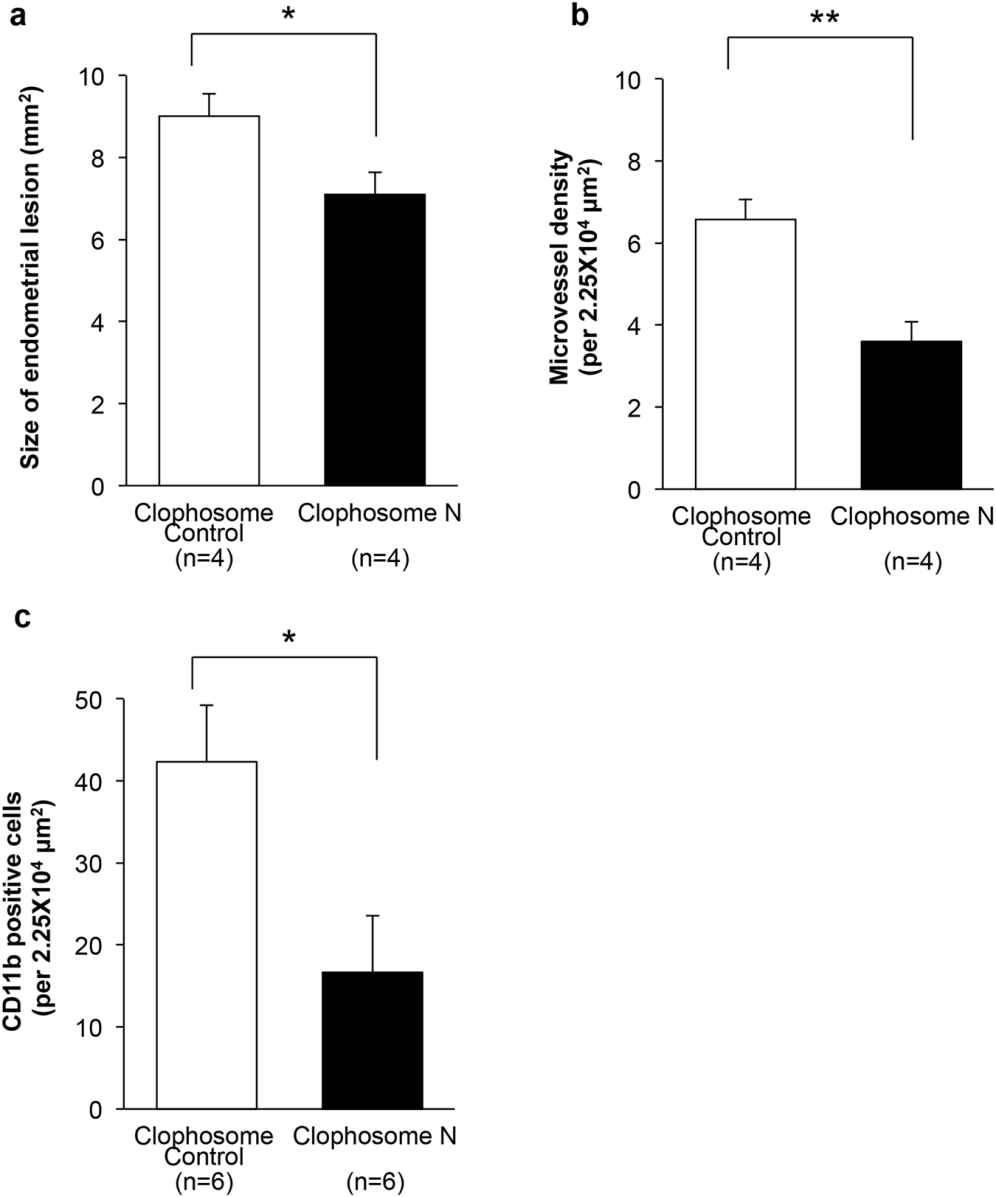
Effect of Clophosome N on growth and angiogenesis in endometrial implants (**a**,**b**) Administration of Clophosome N (0.2 ml/mouse, intraperitoneally) suppressed growth (**a**) and angiogenesis (**b**) in endometrial tissues in the WT → WT at Day 14. Data are expressed as the mean ± SEM (n = 4 in each group). *P < 0.05 and **P < 0.01 compared with the control (Student’s *t*-test). (**c**) The CD11b + cell population was markedly reduced after Clophosome N treatment. Data are expressed as the mean ± SEM (n = 6 in each group). *P < 0.05 compared with the control (Student’s *t*-test).

When we depleted macrophages using Clophosome N, we found that both endometrial tissue growth (Clophosome Control: 9.01 ± 0.54 mm^2^ vs. Clophosome N: 7.01 ± 0.13 mm^2^, P = 0.036, Fig. 5a) and angiogenesis (Clophosome Control: 6.63 ± 0.58 3/2.25 × 10^4^ μm^2^ vs. Clophosome N: 3.58 ± 0.58 3/2.25 × 10^4^ μm^2^, P = 0.0050, Fig. 5b) were significantly suppressed. Taken together, these results suggest that the accumulation of VEGFR1-expressing cells, possibly macrophages from the bone marrow, is the key event that facilitates both growth and angiogenesis of endometrial tissues.

### Flow cytometric analysis for CD11b^+^ cells in the endometrial implants

**Figure 6:**
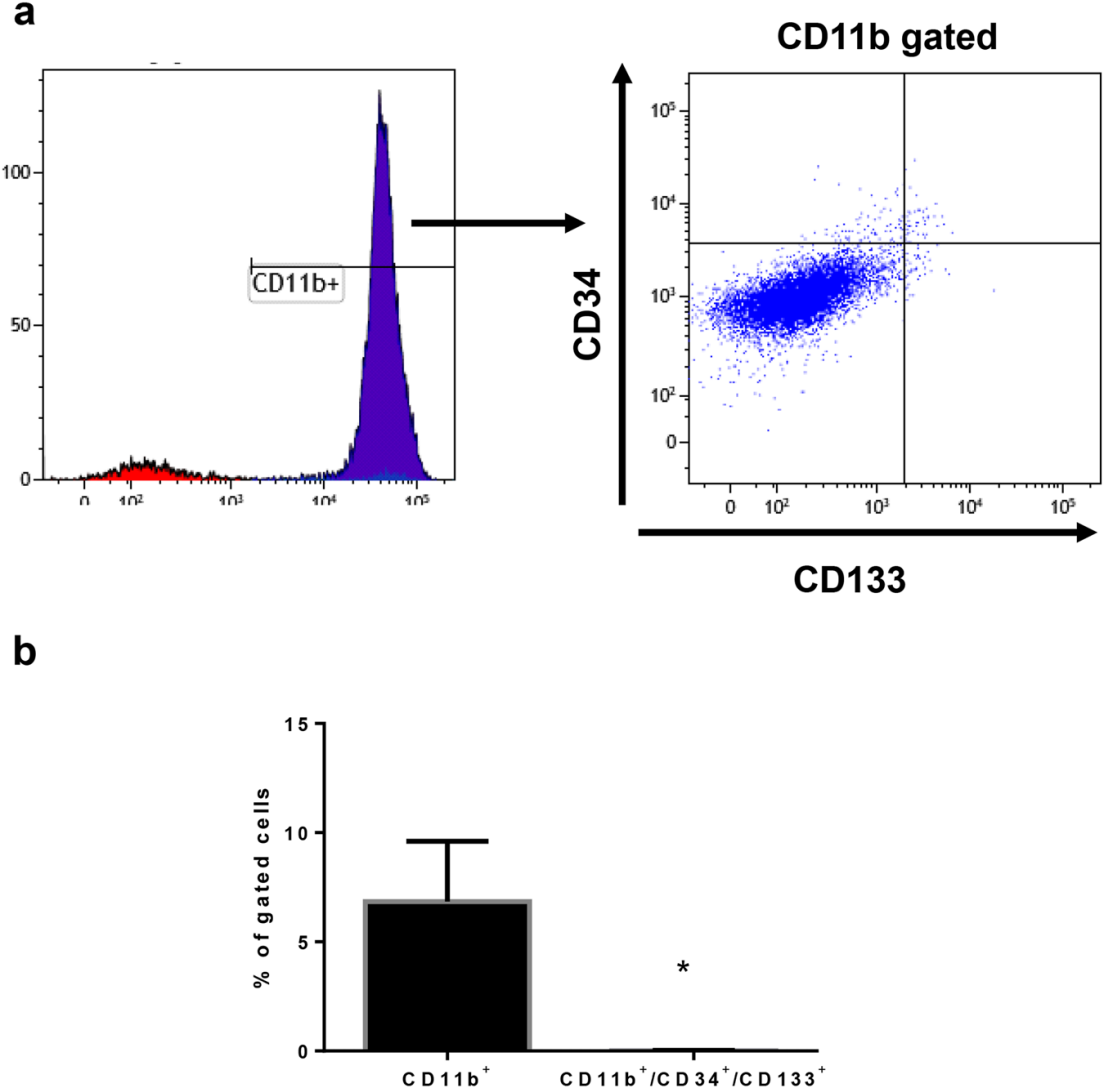
Flow cytometric analysis for CD11b^+^ cells in the endometrial implants (**a**) Flow cytometric dot plots analysis for CD11b^+^ cells and CD34^+^/CD133^+^ cells isolated from the implants with WT → WT at Day 14. (**b**) The percentage of CD11b^+^ cells and CD11b^+^/CD34^+^/CD133^+^ cells. Data are expressed as the mean ± SEM (n = 4 in each group). *P < 0.05 compared with the CD11b^+^ cells (Student’s *t*-test).

Although our data suggested that newly formed blood vessels were derived from the pre-existing blood vessels in the implants, it has been suggested that CD11b^+^ mononuclear cells give rise to endothelial cell-like colonies^36,37^. Therefore, we further examined whether or not CD11b^+^ cells have a profile of endothelial progenitor cells. To this aim, we determined whether CD11b^+^ cells in the implants are positive for markers for endothelial progenitor cells including CD133 and CD34 using flow cytometry analysis. Flow cytometry analysis revealed that the percentage of CD11b^+^ cells in the implants were 6.8 ± 2.2%, while the percentage of CD11b^+^/CD133^+^/CD34^+^ were few (0.02 ± 0.01%, P = 0.0079) (Fig. 6), suggesting that CD11b^+^ macrophages do not have an endothelial progenitor cell profile.

### Molecules that interact with VEGFR1 in endometrial implants

**Figure 7:**
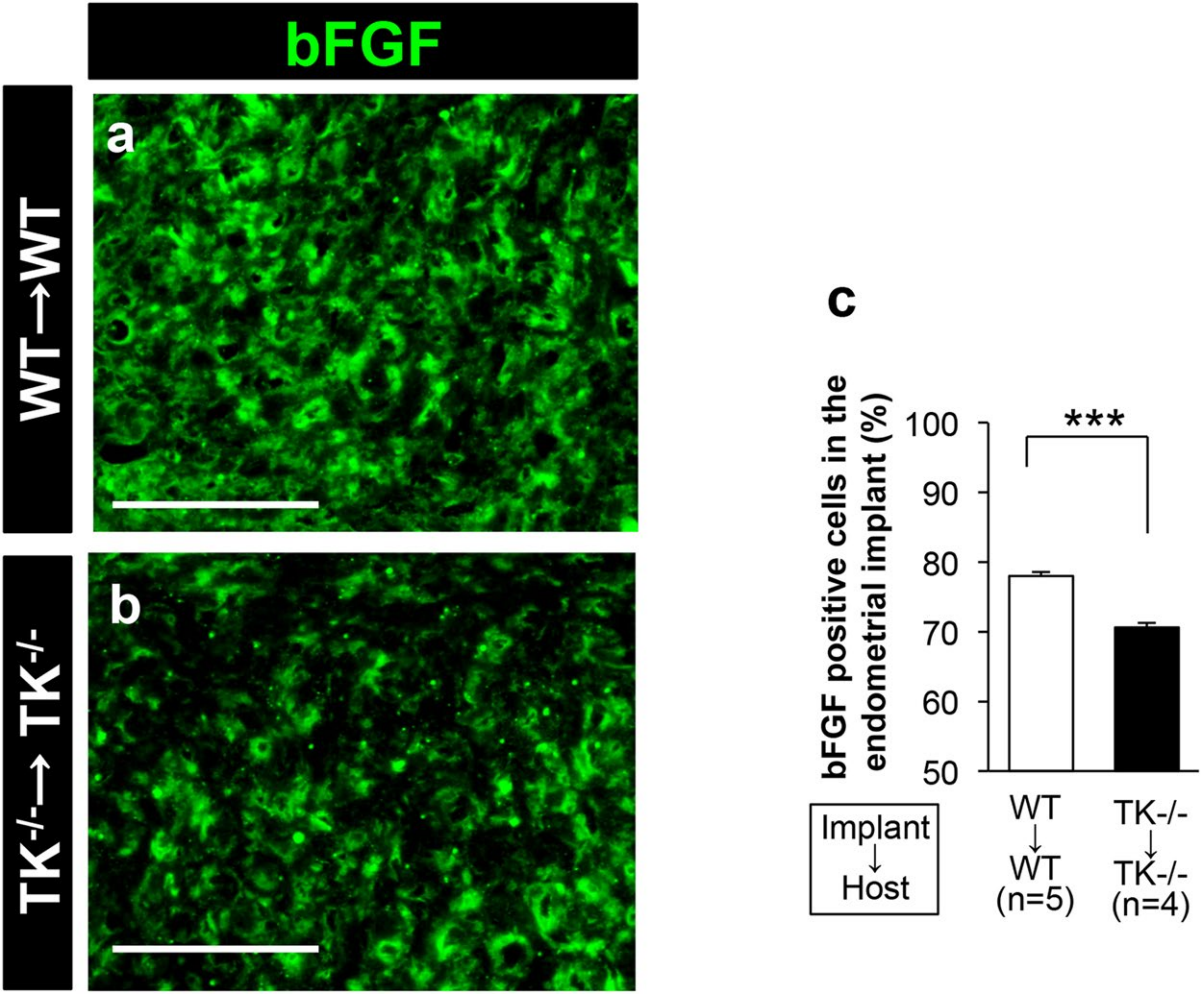
Lack of VEGFR1 signaling suppresses bFGF expression in endometrial tissues (**a**,**b**) Expression of bFGF in endometrial implants from WT → WT (a) and TK^−/−^ → TK^−/−^ (b) at Day 14. Scale bars, 50 μm. (**c**) Number of bFGF^+^ cells in the endometrial implants at Day 14. Data are expressed as the mean ± SEM (n = 4–5 mice). ***P < 0.001 (Student’s *t*-test).

Finally, we attempted to identify the downstream molecules regulated by VEGFR1 signaling in implanted endometrial tissues). Expression of VEGF-A, a ligand for VEGFR1, was induced to a similar extent in endometrial tissues in the WT → WT (7.04 ± 0.66 × 10^−3^) and TK^−^/→ TK^−/−^ (6.00 ± 0.60 × 10^−3^) (Supplementary Fig. S3a). Expression of VEGF-A was observed in CD11b^+^ and S100A4^+^ cells (Supplementary Fig. S4). When we examined other growth factors that regulate angiogenic responses (Supplementary Fig. S3), we found that expression of bFGF in the TK^−/−^ → TK^−/−^ was significantly lower than that in the WT → WT (WT → WT: 3.36 ± 0.18 × 10^−3^ *vs*. TK^−/−^ → TK^−/−^: 2.54 ± 0.13 × 10^−3^, P = 0.0006) (Supplementary Fig. 3b). In addition, CD11b^+^ and S1004A^+^ cells were also positive for bFGF (Supplementary Fig. S5). The accumulation of bFGF^+^ cells in implanted endometrial tissues was significantly lower in the TK^−/−^ → TK^−/−^ than in the WT → WT (WT → WT: 78.0 ± 0.6% *vs*. TK^−/−^ → TK^−/−^: 70.6 ± 0.6%, P < 0.0001) (Fig. 7).

**Figure 8:**
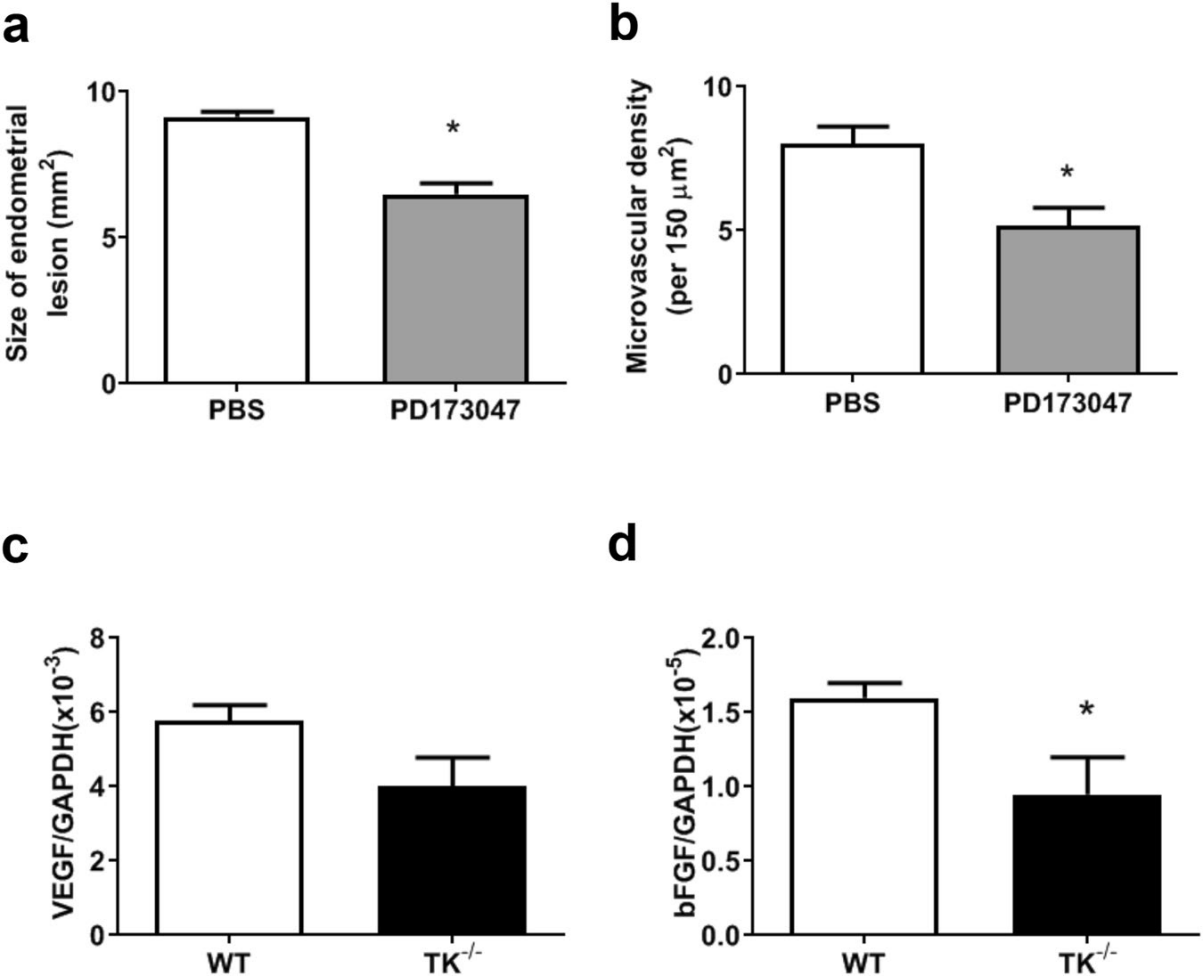
Effect of FGF inhibition in growth and angiogenesis in implants and FGF induction by PlGF in macrophages (**a**,**b**) FGF inhibition with PD173047 reduced growth (**a**) and angiogenesis (**b**) in endometrial implants from WT → WT at Day 14. Data are expressed as the mean ± SEM (n = 8 mice). *P < 0.05 (Student’s *t*-test t). (**c**,**d**) mRNA expression of VEGF (**c**) and bFGF (**d**) in isolated macrophages from WT and TK^−/−^ mice. Isolated bone marrow macrophages were stimulated with PlGF. Data are expressed as the mean ± SEM (n = 4 mice). *P < 0.05 (Student’s *t*-test t).

To elucidate the role of bFGF in the development of endometrial tissue and angiogenesis in endometriosis, we treated mice with FGFR inhibitor, PD173047 (Fig. 8a,b). Treatment with PD173047 significantly reduced size of endometriosis (PBS: 8.81 ± 0.20 mm^2^ *vs*. PD173047: 6.76 ± 0.33 mm^2^, P < 0.0001, Fig. 8a) and microvascular density (PBS: 8.00 ± 0.61/2.25 × 10^4^ μm^2^ *vs*. PD173047: 5.16 ± 0.63/2.25 × 10^4^ μm^2^, P = 0.0063, Fig. 8b) in recipient WT with endometrial implants from WT mice. To further examine whether or not VEGFR1-expressing macrophages produce bFGF, isolated bone marrow-derived macrophages from WT and TK^−/−^ mice were stimulated with PlGF, a specific agonist for VEGFR1. In *in vitro* study, the expression of bFGF in response to PlGF in bone marrow-derived WT-macrophages was higher than that from bone marrow-derived TK^−/−^macrophages (WT: 1.60 ± 0.10 × 10^−5^ *vs*. TK^−/−^: 0.93 ± 0.27 × 10^−5^, P = 0.03) (Fig. 8d). However, there was no significant difference in VEGF expression between the genotype (WT: 5.78 ± 0.42 × 10^−3^ *vs*. TK^−/−^: 4.03 ± 0.76 × 10^−3^, P = 0.09) (Fig. 8c).

These data suggest that VEGFR1 signaling increases bFGF expression, which then modulates growth and angiogenesis in endometrial tissues.

## Discussion

In the present study, we showed that VEGF was a key regulator of angiogenesis in endometrial tissues. Cross transplantation experiments using TK^−/−^ and WT mice revealed that VEGFR1 signaling in host-derived cells in the implants, played a role in both growth and angiogenesis. Accumulation of VEGFR1^+^ macrophages from the host bone marrow was the key driver of growth and angiogenesis in the endometrial implants. The results obtained suggested that blocking VEGFR1 signaling will be a promising strategy for the treatment of endometriosis.

Using genetically engineered mice, we recently reported that angiogenic responses in mice with hindlimb ischemia were enhanced by VEGFR1 signaling but not by VEGFR2 signaling^18^, suggesting that the receptors responsible for signaling during ischemia and endometriosis were similar. The mechanisms underlying the establishment of endometriotic lesions are not fully understood; however, there is no doubt that the long-term survival and proliferation of these lesions are crucially dependent on the formation of new blood vessels, which guarantee the supply of oxygen and essential nutrients^9,38–40^. Endometriotic lesions are typically characterized by dense vascularization^1,41,42^. Cross transplantation of endometrial tissues isolated from genetically engineered mice is a very useful strategy for clarifying the cellular origin and tissue-specific functions of proangiogenic factors. Our data indicated that VEGFR1 signaling in host-derived cells is responsible for angiogenesis and growth in the endometrial tissues (Figs 2, 4 and Supplementary Fig. S2). We had previously reported that VEGFR1-expressing macrophages that accumulate in damaged tissues facilitate tissue repair and vessel reconstruction^43^. VEGFR1 TK^−/−^ bone marrow chimera mice exhibit delayed healing and vessel reconstruction after tissue damage, suggesting the VEGFR1-positive macrophages recruited from the bone marrow play a significant role in this process^43^. Here, we also indicated that bone marrow-derived macrophages expressing VEGFR1 in host drove both angiogenic responses in the implants and the growth of endometrial tissues (Fig. 4). It is frequently reported that macrophages increase angiogenesis under pathological conditions^44–46^. Consistent with this, we found that the macrophages play a significant role in the development of endometriosis (Fig. 4). Immunofluorescence suggests that VEGF-A is produced by macrophages and fibroblasts (Supplementary Fig. S4). Because VEGF-A induces chemotaxis in peritoneal macrophages through VEGFR1-mediated mechanisms^14^, and VEGFR1 mediates monocyte/macrophage infiltration to local inflammatory sites^16,18,43^, VEGF-A released from macrophages and fibroblasts recruits macrophages expressing VEGFR1 to develop the endometrial tissue. Taken together, VEGFR1-expressing macrophages recruited via VEGF-A/VEGFR1 signaling promoted angiogenesis in the endometrial implants, leading to the maintenance and growth of ectopic endometrial tissues.

The current study demonstrated that VEGFR1^+^ cells express S100A4, which is an S100 protein that is known to be a specific marker for fibroblasts^35^. We previously reported that fibroblasts recruited from the bone marrow accumulate in stromal tissues during up-regulated tumor-associated angiogenesis and tumor growth^22^; however, the host-derived S1004A^+^ cell population in the implants in the bone marrow transplantation experiments was smaller than the host-derived CD11b^+^ population. These results indicate that S1004A^+^ fibroblasts play a minor role in promotion of angiogenesis and development of endometriosis.

We were surprised that CD31^+^ vascular endothelial cells in the implants were not GFP^+^; this was the case even in GFP transgenic WT bone marrow chimera mice (Fig. 3b). We also demonstrated that accumulated CD11b^+^ macrophages in the implants displayed no property of endothelial progenitor cells. This suggests that post-natal vasculogenesis may play only a minor role during the development of endometriosis. Although the mechanisms underlying the establishment of endometriotic lesions are unclear, it is possible that vasculogenesis plays a role in endometriosis; however, a previous study shows that 13% (at most) of endothelial cells in a mouse endometriosis model were derived from the bone marrow^47^. The results of the current study suggest that the majority of blood vessels in the implants grew in a macrophage-dependent manner rather than by vasculogenesis. Thus, our results are consistent with those in the above report showing that vasculogenesis is not the main driver of blood vessel formation in endometrial tissues. Our results also suggest that accumulated host-derived macrophages promote the formation of new blood vessels from the preexisting tissues in the implants.

We also found that VEGFR1 signaling was a major determinant of neovascularization in endometrial tissues. Blockade of VEGF signaling with a soluble VEGF receptor or an affinity-purified anti-VEGF antibody is an effective treatment for endometriosis in nude mice^48^. However, as shown in Supplementary Fig. S3, endometrial tissues not only express VEGF but also various other growth factors. Among these, we found that bFGF expression was dependent upon VEGFR1 (Supplementary Fig. S3, and Fig. 7). Thus, it is plausible that the development of new blood vessels in endometriotic lesions is critically dependent on the interaction between multiple signaling molecules including bFGF. bFGF was reported to drive angiogenesis in endometrial tissues^49,50^. Consistent with these observations, our data showed that FGF receptor inhibition suppressed the growth of endometrial tissue and angiogenesis. These taken together suggested that growth factors including VEGF and bFGF interacted with VEGFR1.

It has been shown that inhibition of VEGFR1 signaling attenuates tumor growth and rheumatoid arthritis through suppressing angiogenesis^51^. Angiogenesis inhibitors including tyrosine kinase inhibitors display a beneficial effect on endometriosis in rodents^52^. Additionally, progesterone derived from ovary induces the expression of VEGF-A, which is a critical factor in the dynamic regulation of the uterine vasculature during postmenstrual repair as well as pregnancy^53^. Furthermore, the degree of preeclampsia is well correlated with increased serum levels of soluble fms-like tyrosine kinase-1 (sFlt-1) in pregnant mothers. Because sFlt-1 would form a molecular barrier against abnormal vascular permeability and abnormal angiogenesis, by trapping VEGF and PlGF, sFlt-1-blocking agents could treat preeclampsia^51^. Because VEGF-A neutralizing antibody and multi-tyrosine kinase VEGFR inhibitor have been widely used in the treatment of cancer for suppressing angiogenesis, VEGFR1 inhibition would be a useful tool for regulation of endometriosis-associated angiogenesis in reproductive aged women.

**Figure 9:**
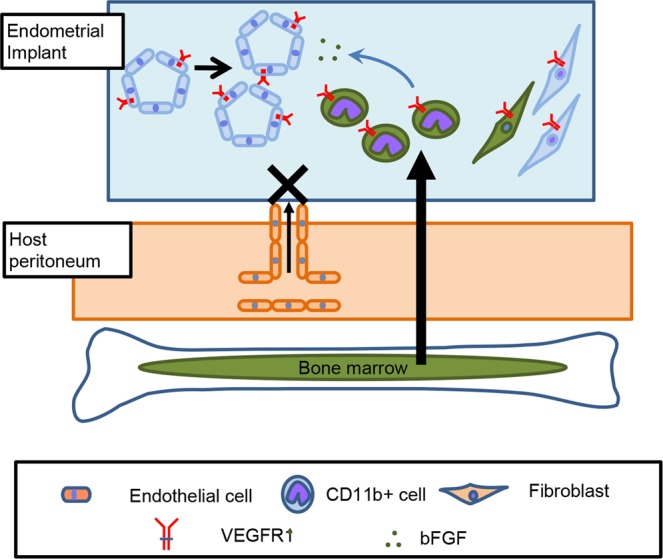
Roles of VEGFR1 signaling that facilitate angiogenesis in endometrial tissues VEGF is a key regulator of growth and angiogenesis in endometrial tissues. Cross transplantation experiments using TK^−/−^ and WT mice revealed that VEGFR1 signaling in the host, or host-derived cells in the implants, played a role in both growth and angiogenesis. The blood vessels in the implants were not derived from the host peritoneum. Immunostaining for VEGFR1 suggested that high numbers of VEGFR1^+^ cells such as macrophages were infiltrated into the endometrial tissues. Accumulation of VEGFR1^+^ macrophages from the host bone marrow was the key driver of angiogenesis in the endometrial implants via secretion of bFGF.

In conclusion, VEGF is a key regulator of growth and angiogenesis in endometrial tissues (Fig. 9). Accumulation of VEGFR1^+^ macrophages from the host bone marrow was the key driver of growth and angiogenesis in the endometrial implants via secretion of bFGF. Taken together, these results suggest that blocking VEGFR1 with antibodies or a small molecule kinase inhibitor will be a promising strategy for the treatment of endometriosis.

## Supplementary information

Supple text

## Data Availability

The datasets generated during and/or analyzed during the current study are available from the corresponding author on reasonable request.
