# Supplementary material for: VEGF Receptor 1-Expressing Macrophages Recruited from Bone Marrow Enhances Angiogenesis in Endometrial Tissues
Source: Sci Rep. 2019 May 7;9:7037. doi: 10.1038/s41598-019-43185-8 (PMC6504918; doi:10.1038/s41598-019-43185-8)
Supplement: Supplementary file 1 — Supple text [file 41598_2019_43185_MOESM1_ESM.pdf]

Supplementary Information

VEGF Receptor 1-Expressing Macrophages Recruited from Bone Marrow Enhances Angiogenesis in Endometrial Tissues

Kazuki Sekiguchi[1,2,3¶], Yoshiya Ito[1,2¶], Kyoko Hattori[1,2,3], Tomoyoshi Inoue[1,2], Kanako Hosono[1,2], Masako Honda[1,2,3], Akiko Numao[3], Hideki Amano[1,2], Masabumi Shibuya[4], Nobuya Unno[3], Masataka Majima[1,2]

¶ These authors contributed equally to this work.

[1]Department of Pharmacology, Kitasato University School of Medicine, [2]Department of Molecular Pharmacology, Graduate School of Medical Sciences, [3]Department of Gynecology, Graduate School of Medical Sciences, Kanagawa, 252-0374, Japan, and [4]Gakubunkan Institute of Physiology and Medicine, Jobu University, Gunma 370-0831, Japan.

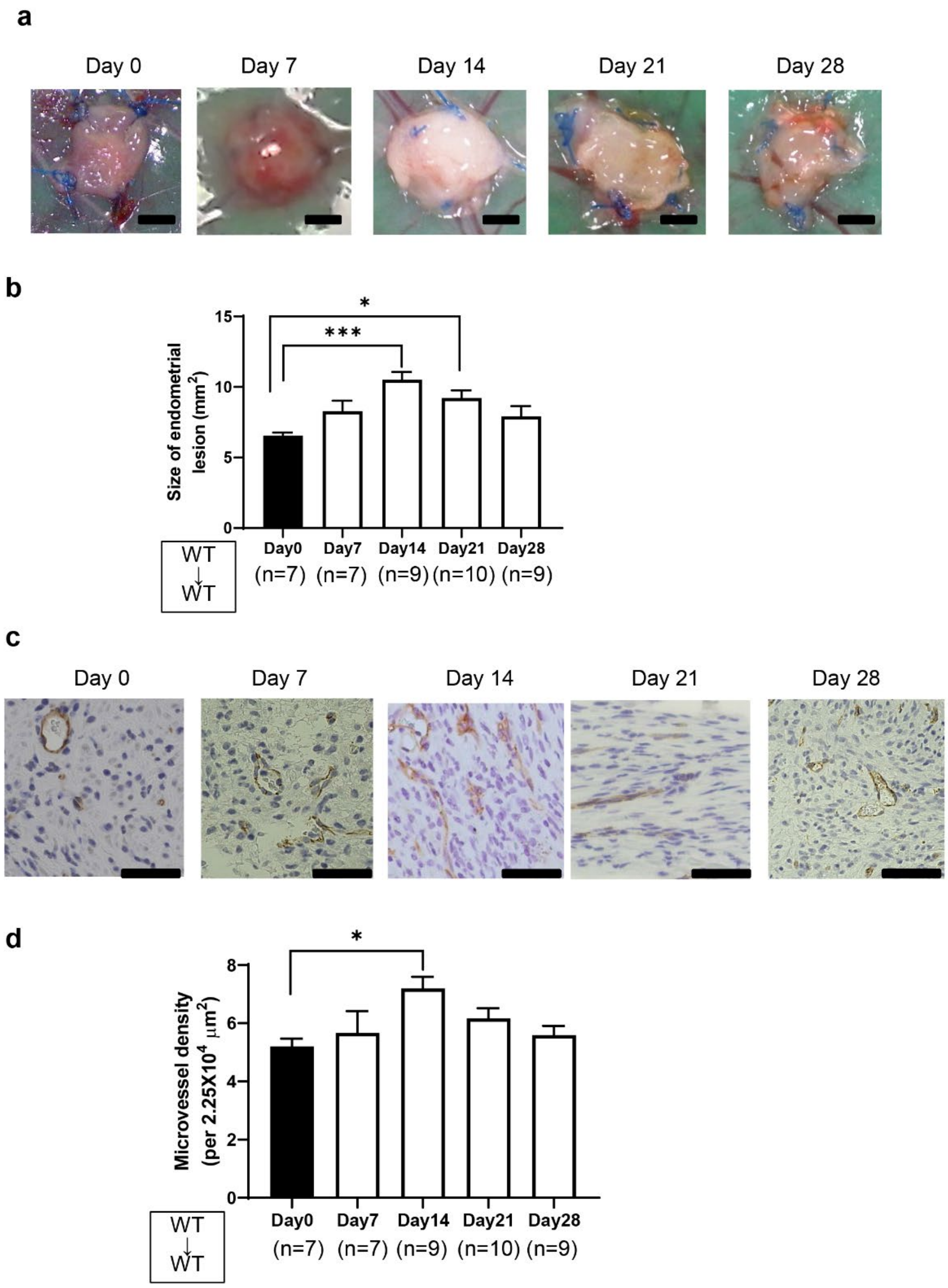
a
Day 0
Day 7
Day 14
Day 21
Day 28
b
Size of endometrial lesion (mm^2)
15
10
5
0
*
***
Day0
Day7
Day14
Day21
Day28
(n=7)
(n=7)
(n=9)
(n=10)
(n=9)
WT
↓
WT
c
Day 0
Day 7
Day 14
Day 21
Day 28
d
Microvessel density (per 2.25X10^4 μm^2)
8
6
4
2
0
*
Day0
Day7
Day14
Day21
Day28
(n=7)
(n=7)
(n=9)
(n=10)
(n=9)
WT
↓
WT

Supplementary Figure S1. Changes in growth and angiogenesis in endometrial tissues

a) Typical appearance of implants after transplantation. Scale bars, 1 mm. b) Temporal changes in implants sizes after transplantation in the WT→WT. Data are expressed as the mean ± SEM (n = 7–10 mice). * $P < 0.05$ and *** $P < 0.001$ (One-way ANOVA). c) Immunohistochemical staining of endometrial implants sections for CD31 expression from the WT→WT. Scale bars, 50 μm. d) Time course of changes in MVD in the endometrial implants in the WT→WT. Data are expressed as the mean ± SEM (n = 7–10 mice). * $P < 0.05$ (One-way ANOVA).

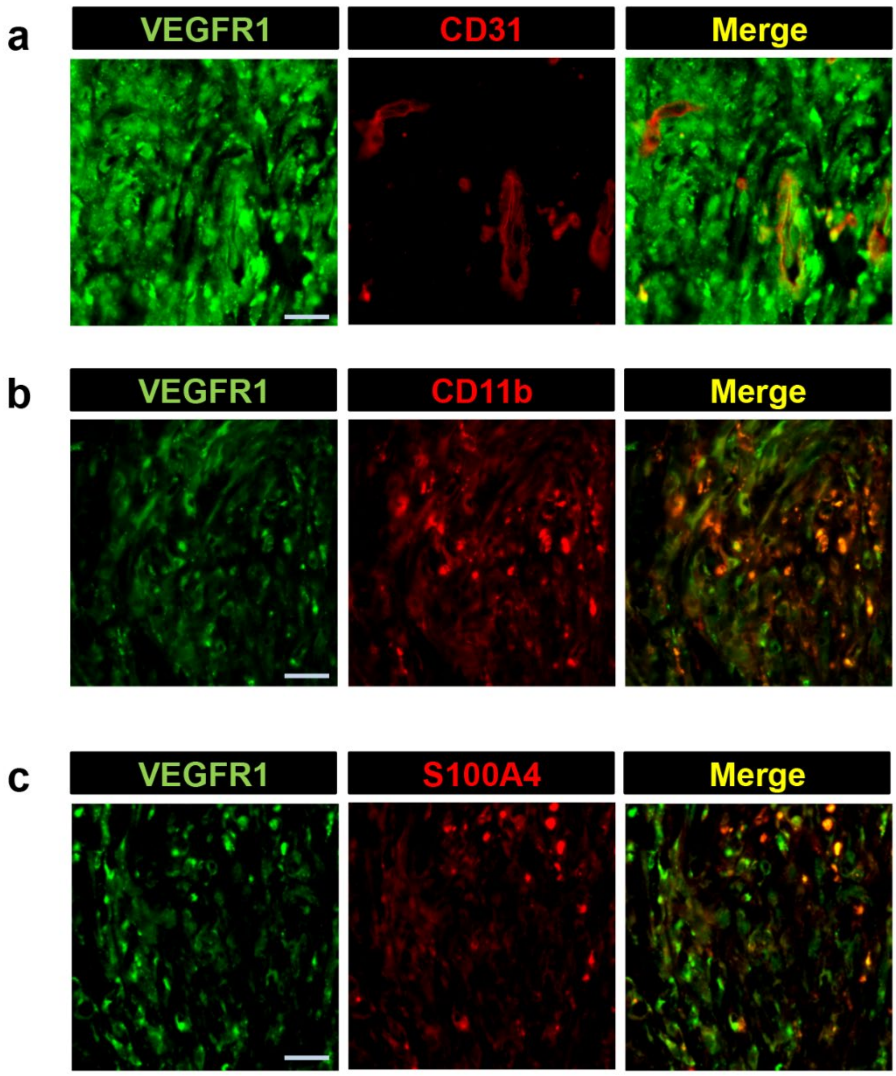

Supplementary Figure S2. VEGFR1$^+$ cells accumulated in endometrial tissues

VEGFR1$^+$ cells in endometrial implants from WT→ WT at Day 14 post-implantation.

a) VEGFR1$^+$ cells; CD31$^+$ cells; and a merged image. Scale bar, 25 µm.

b) VEGFR1$^+$ cells; CD11b$^+$ cells; and a merged image. Scale bar, 25 µm.

c) VEGFR1$^+$ cells; S100A4$^+$ cells; and a merged image. Scale bar, 25 µm.

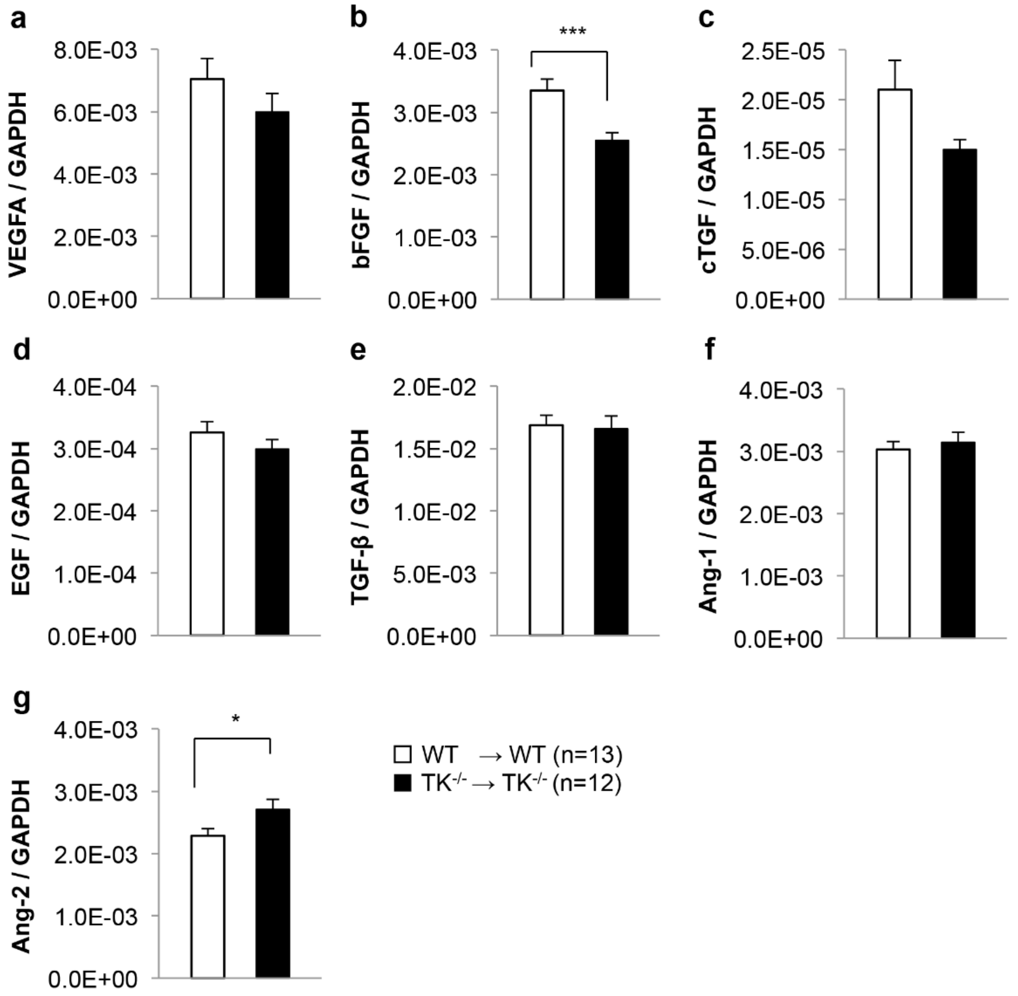

Supplementary Figure S3. Levels of other proangiogenic factors expressed in endometrial implants

a–g) cDNA was generated from endometrial implants from WT→WT and $TK^{-/-}$→$TK^{-/-}$ at Day 14 post-implantation. Levels of VEGF-A (a), bFGF (b), CTGF (c), EGF (d), TGF-β (e), Ang-1 (f), and Ang-2 (g) mRNA were measured by real-time PCR and expressed relative to GAPDH. Data are expressed as the mean ± SEM (n = 12−13 mice). * P < 0.05 and *** P < 0.001 (Student's *t*-test).

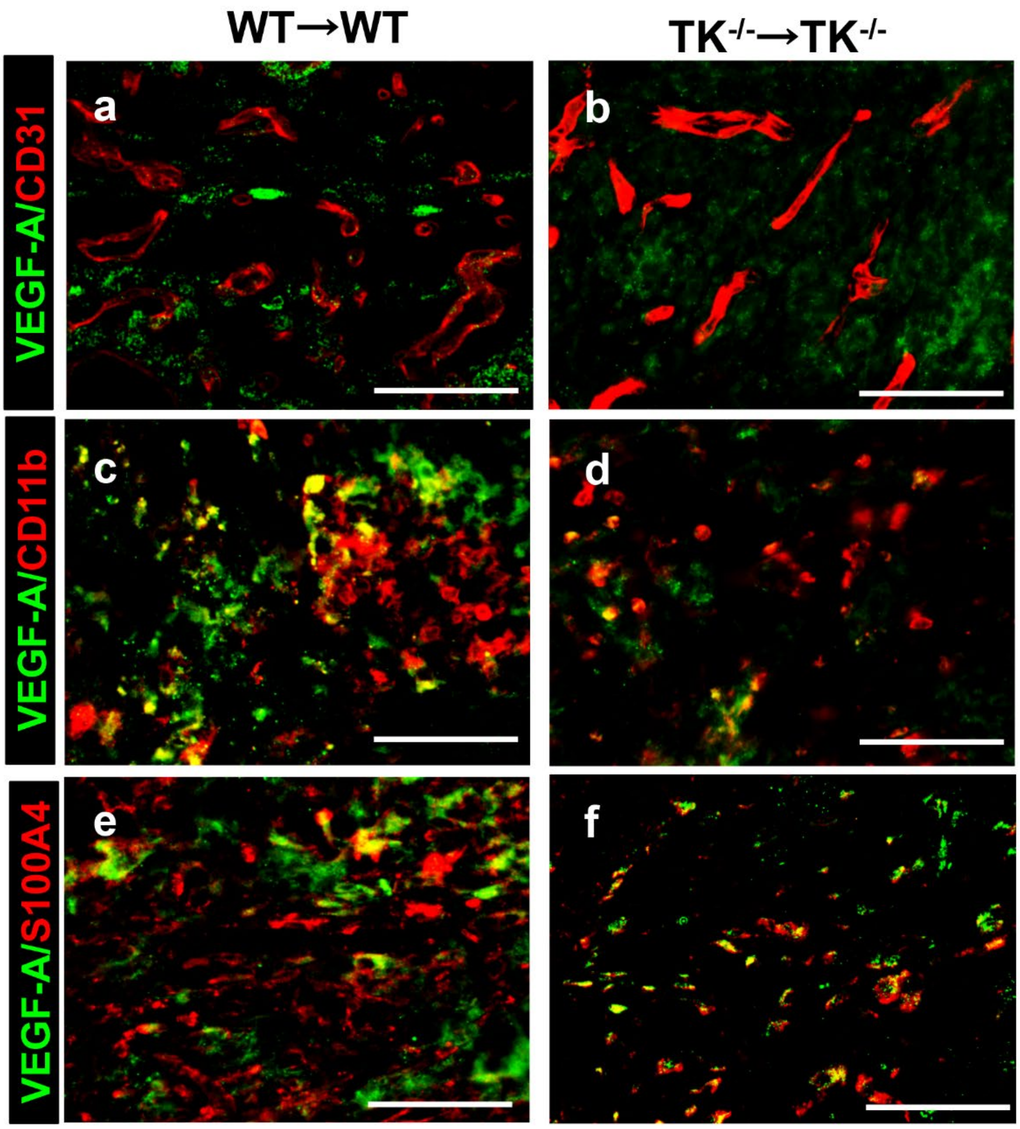

Supplementary Figure S4. VEGF-A expression in endometrial implants

Double immunostaining for VEGF-A and CD31 (a,b) , CD11b (c,d), or S100A4 (e,f) in the WT→WT and $TK^{-/-}$→$TK^{-/-}$, respectively at Day 14 post-implantation. Scale bars, 50 μm.

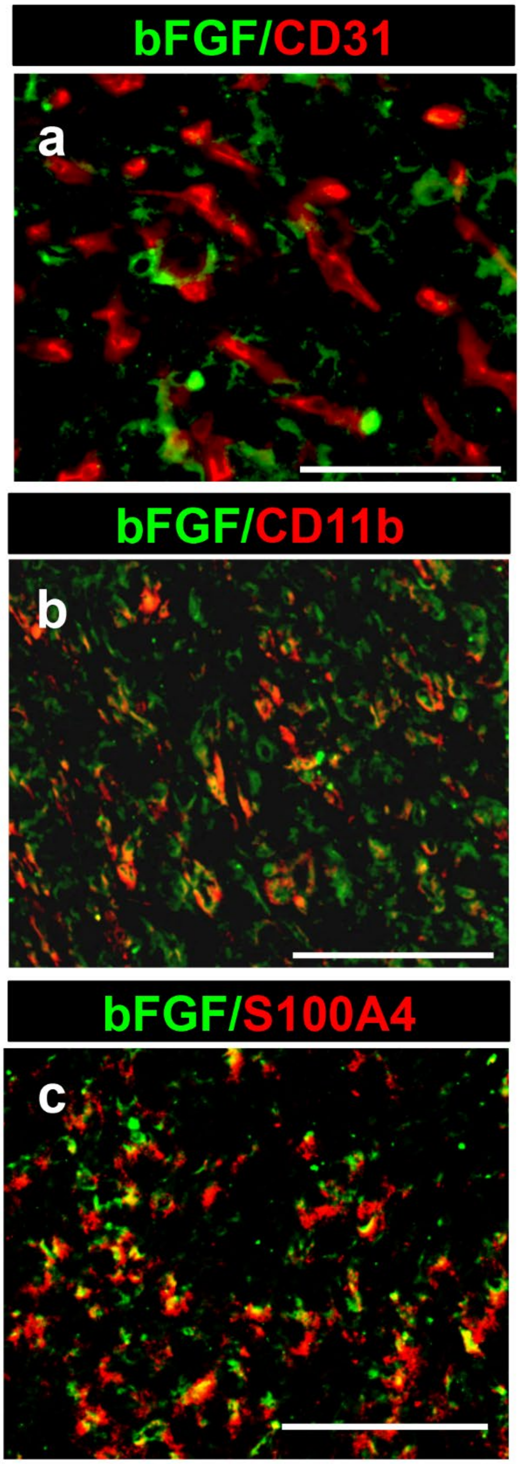

Supplementary Figure S5. bFGF$^+$ cells accumulate in endometrial implants

The distribution of bFGF$^+$ cells was examined in endometrial implants from WT→WT at Day 14. **a-c)** bFGF$^+$ cells co-stained with an anti-CD11b antibody (b) or anti-S100A4 antibody (c), but not with an anti-CD31 antibody (a). Merged images. Scale bars, 50 μm.
